# Collapse of the native structure caused by a single amino acid exchange in human NAD(P)H:quinone oxidoreductase

**DOI:** 10.1111/febs.12975

**Published:** 2014-09-19

**Authors:** Wolf-Dieter Lienhart, Venugopal Gudipati, Michael K. Uhl, Alexandra Binter, Sergio A. Pulido, Robert Saf, Klaus Zangger, Karl Gruber, Peter Macheroux

**Affiliations:** 1 Institute of Biochemistry, Graz University of Technology, Austria; 2 Institute of Molecular Biosciences, University of Graz, Austria; 3 Institute of Chemistry, University of Graz, Austria; 4 Institute of Chemistry and Technology of Materials, Graz University of Technology, Austria

**Keywords:** antioxidant defense, cancer, flavin, quinones, single-nucleotide polymorphism

## Abstract

Human NAD(P)H:quinone oxidoreductase 1 (NQO1) is essential for the antioxidant defense system, stabilization of tumor suppressors (e.g. p53, p33, and p73), and activation of quinone-based chemotherapeutics. Overexpression of NQO1 in many solid tumors, coupled with its ability to convert quinone-based chemotherapeutics into potent cytotoxic compounds, have made it a very attractive target for anticancer drugs. A naturally occurring single-nucleotide polymorphism (C609T) leading to an amino acid exchange (P187S) has been implicated in the development of various cancers and poor survival rates following anthracyclin-based adjuvant chemotherapy. Despite its importance for cancer prediction and therapy, the exact molecular basis for the loss of function in NQO1 P187S is currently unknown. Therefore, we solved the crystal structure of NQO1 P187S. Surprisingly, this structure is almost identical to NQO1. Employing a combination of NMR spectroscopy and limited proteolysis experiments, we demonstrated that the single amino acid exchange destabilized interactions between the core and C-terminus, leading to depopulation of the native structure in solution. This collapse of the native structure diminished cofactor affinity and led to a less competent FAD-binding pocket, thus severely compromising the catalytic capacity of the variant protein. Hence, our findings provide a rationale for the loss of function in NQO1 P187S with a frequently occurring single-nucleotide polymorphism.

## Introduction

NAD(P)H:quinone oxidoreductase 1 (NQO1; EC 1.6.99.2) is a dimeric (61.7 kDa) human cytosolic FAD-dependent enzyme catalyzing the two-electron reduction of intracellular quinones to hydroquinones. NQO1 is an essential component of the antioxidant defense system, preventing the formation of potentially harmful semiquinone radicals [1]. Additionally, NQO1 stabilizes several tumor suppressors (p33^ING1b^, p53, and p73), thereby exerting an antineoplastic effect [2–5], and also activates quinone-based chemotherapeutics by reducing the quinone pharmacophore to a cytotoxic hydroquinone form [6]. It was recently shown that NQO1 maintains mitochondrial integrity as a part of the p62–Keap1–Nrf2–Nqo1 cascade that has evolved to prevent mitochondrial dysfunction, thus attenuating the rate of aging in vertebrates [7].

A frequent single-nucleotide polymorphism (SNP) in the *NQO1* gene (on human chromosome 16q22.1), whereby C609 is changed to T, results in the replacement of Pro187 by serine in the protein [8]. The frequency of the NQO1*2 homozygous genotype was estimated to be between 4% and 20%, depending on the ethnic group, with the highest prevalence being seen in Asian populations [9]. The NQO1*2 genotype is prevalent in individuals (> 25%) who are susceptible to increased benzene hematotoxicity and acute myelogenic leukemia. In addition, it appears to be linked to poor survival rates of women with breast cancer after anthracycline-based adjuvant chemotherapy [10–12]. It was proposed that the occurrence of the NQO1*2 genotype is a prognostic and predictive marker for breast cancer [10].

Despite its importance for cancer prediction and therapy, the exact structural and molecular basis for the loss of function in NQO1 P187S is currently unknown. The site of amino acid exchange (P187S) is neither near the FAD-binding active site of the enzyme nor near the NAD(P)H-binding site. Therefore, it was speculated that the proline to serine replacement leads to local perturbation of a central β-sheet, reducing the affinity of FAD, and thus lowering catalytic activity [13]. It was also proposed that FAD acts as a chemical chaperone, maintaining the properly folded state of NQO1 [14]. Our findings establish that the amino acid replacement destabilizes the native fold of the enzyme, thus contradicting previous assumptions proposed to rationalize the loss of function in NQO1 P187S.

## Results and Discussion

**Fig. 1:**
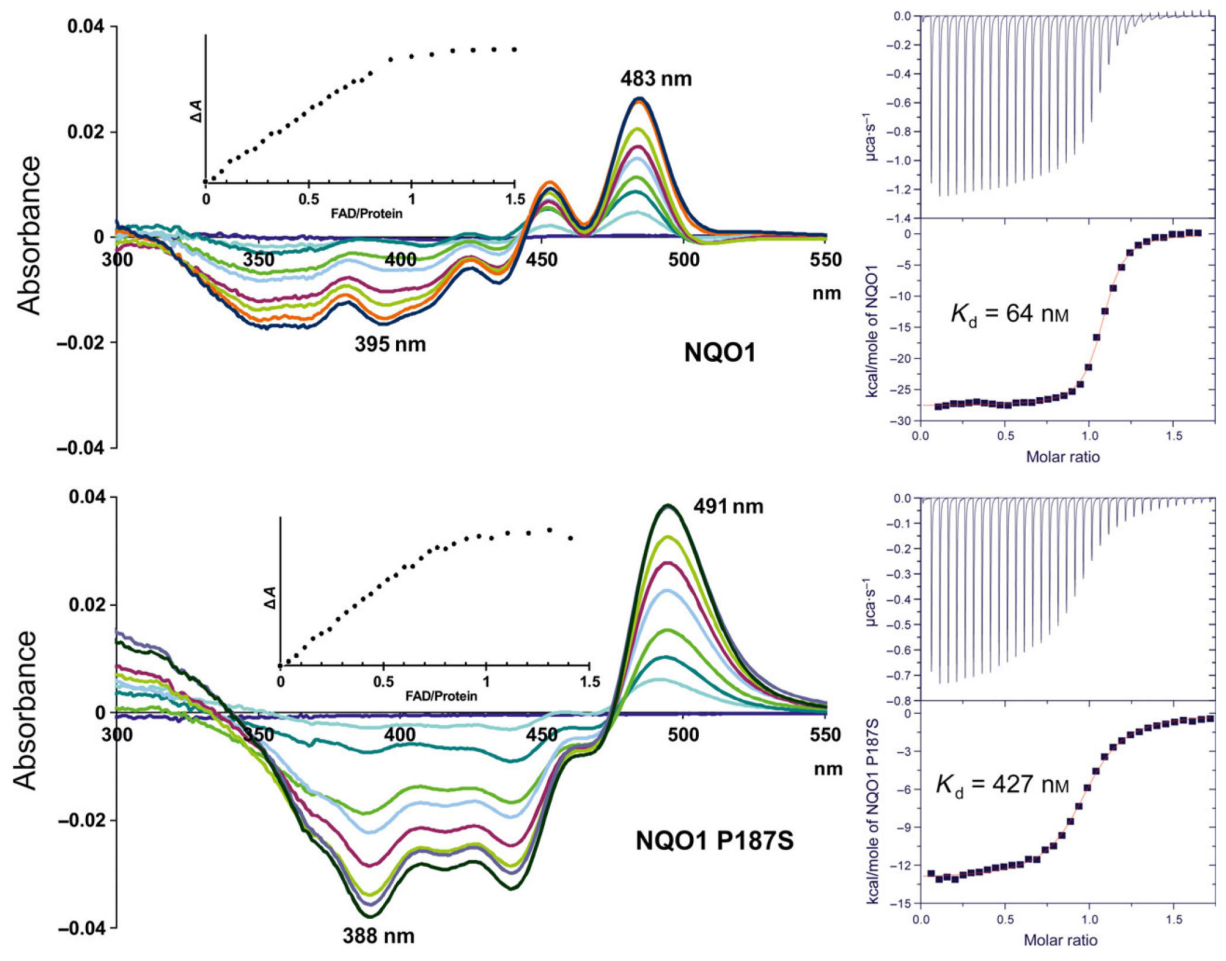
The binding mode and affinity of FAD for NQO1 P187S are different from those for NQO1. Left diagrams: UV–visible difference absorption spectra. Representative difference absorption spectra obtained from the difference titration of apo-NQO1 and apo-NQO1 P187S with the FAD cofactor in the spectral range of 300–550 nm are shown. The spectra were recorded after the addition of 0 μL (black), 6 μL (red), 12 μL (light green), 18 μL (orange), 24 μL (light blue), 30 μL (magenta), 36 μL (blue), 45 μL (green) and 60 μL (light brown) of FAD stock solution (1 mm). Insets: the spectral changes were monitored by plotting the sum of the absolute value at 435 nm and 483 nm (NQO1) or 437 nm and 491 nm (NQO1 P187S) of all of the spectra against the FAD/protein ratio. The data indicate a binding ratio of one FAD per subunit for both NQO1 and NQO1 P187S. Right diagrams: ITC. NQO1 (top right) and NQO1 P187S (bottom right) were titrated into an FAD solution. The upper panel in each diagram shows the time-dependent release of heat during the titration (exothermic). Peak integrals as a function of the FAD/protein molar ratio are shown in the bottom panel of each diagram.

In view of the important cellular functions of NQO1 and the high frequency of the NQO1*2 genotype, we studied the biochemical and structural properties of NQO1 P187S in comparison with NQO1. During purification of the recombinant NQO1 proteins by Ni^2+^–nitrilotriacetic acid affinity and size exclusion chromatography, we noticed that NQO1 P187S showed partial depletion of the FAD cofactor, indicating that it had lower cofactor-binding affinity than NQO1. Therefore, we prepared the apo-forms of NQO1 and NQO1 P187S, and studied binding of the FAD cofactor to the apo-forms by monitoring difference absorption changes. As shown in Fig. 1 (left diagrams), the spectral perturbations observed for NQO1 and NQO1 P187S showed clear differences in their absorption minima and maxima, as well as their isosbestic points. However, both titrations produced sharp endpoints (Fig. 1, insets), indicating that the dissociation constants of FAD binding are below the micromolar range for both NQO1 and NQO1 P187S. As the dissociation constants for both proteins are in the same range, we assumed that reduced activity of NQO1 P187S cannot be explained solely by the loss of FAD, as suggested in previous studies [14]. For the accurate determination of dissociation constants, FAD was titrated with apo-NQO1 and apo-NQO1 P187S in a microcalorimeter. These isothermal titration microcalorimetry (ITC) measurements showed that each protomer of the dimeric protein has a single FAD-binding site, albeit NQO1 P187S showed an increase in the dissociation constant (*K*_d_) by a factor of 7 as compared with NQO1 (*K*_d_ = 64 ± 23 nm and *K*_d_ = 428 ± 90 nm for NQO1 and NQO1 P187S, respectively; Fig. 1, right diagrams). Thus, our experiments show that FAD binding is affected not only qualitatively in terms of the mode of physical interactions, but also quantitatively (i.e. lower affinity).

Furthermore, rapid reaction measurements demonstrated that the reductive half-reaction of the catalytic cycle is severely affected in NQO1 P187S. The bimolecular rate constants of NQO1 P187S were decreased by factors of 300 and 70 for NADH and NADPH, respectively (Table 1). On the other hand, the oxidative half-reaction, i.e. reduction of a quinone substrate (e.g. benzoquinone or menadione) by the reduced FAD cofactor, was still very rapid in NQO1 P187S and was complete within the dead time of the stopped-flow instrument (~ 5 ms). As the reductive half-reaction is the rate-limiting step in the catalytic cycle, the lower rate of reduction leads directly to a decrease in catalytic turnover in NQO1 P187S. Hence, it is highly probable that NQO1 P187S will metabolize cellular quinones, radical oxygen species and prodrugs at a much lower rate than that of NQO1. In this context it should be noted that the much lower rate of reduction strongly indicates that the FAD-binding site is catalytically less competent, corroborating the conclusion that the mode of FAD binding is different in NQO1 P187S.

**Fig. 2:**
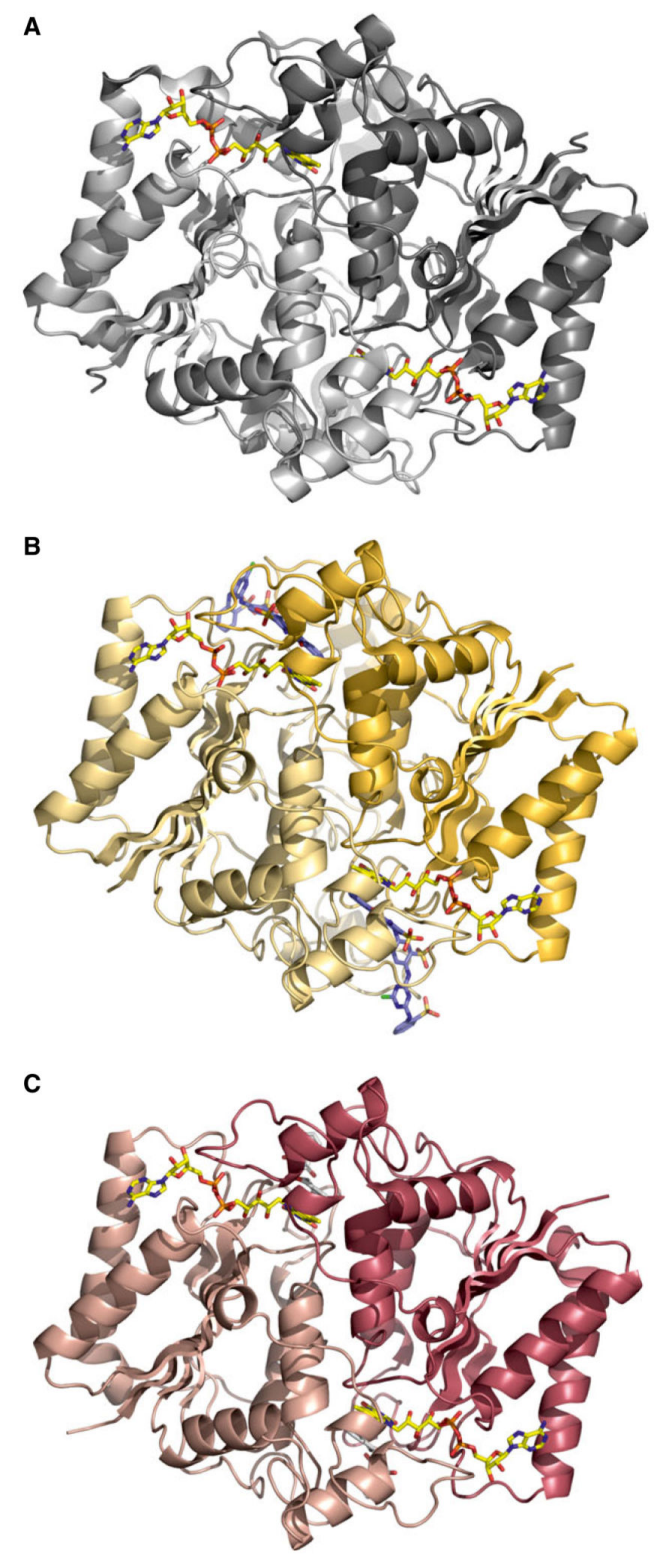
Comparison of the X-ray crystallographic dimeric structures of NQO1, NQO1 P187S, and the truncated form of NQO1 P187S. (A) X-ray crystal structure of NQO1 (PDB code: 1d4a). (B, C) X-ray crystal structures of NQO1 P187S and the truncated form of NQO1 P187S (50 amino acids at the C-terminus), respectively. FAD cofactor (A–C) and Cibacron blue (B) are shown as stick models.

**Fig. 3:**
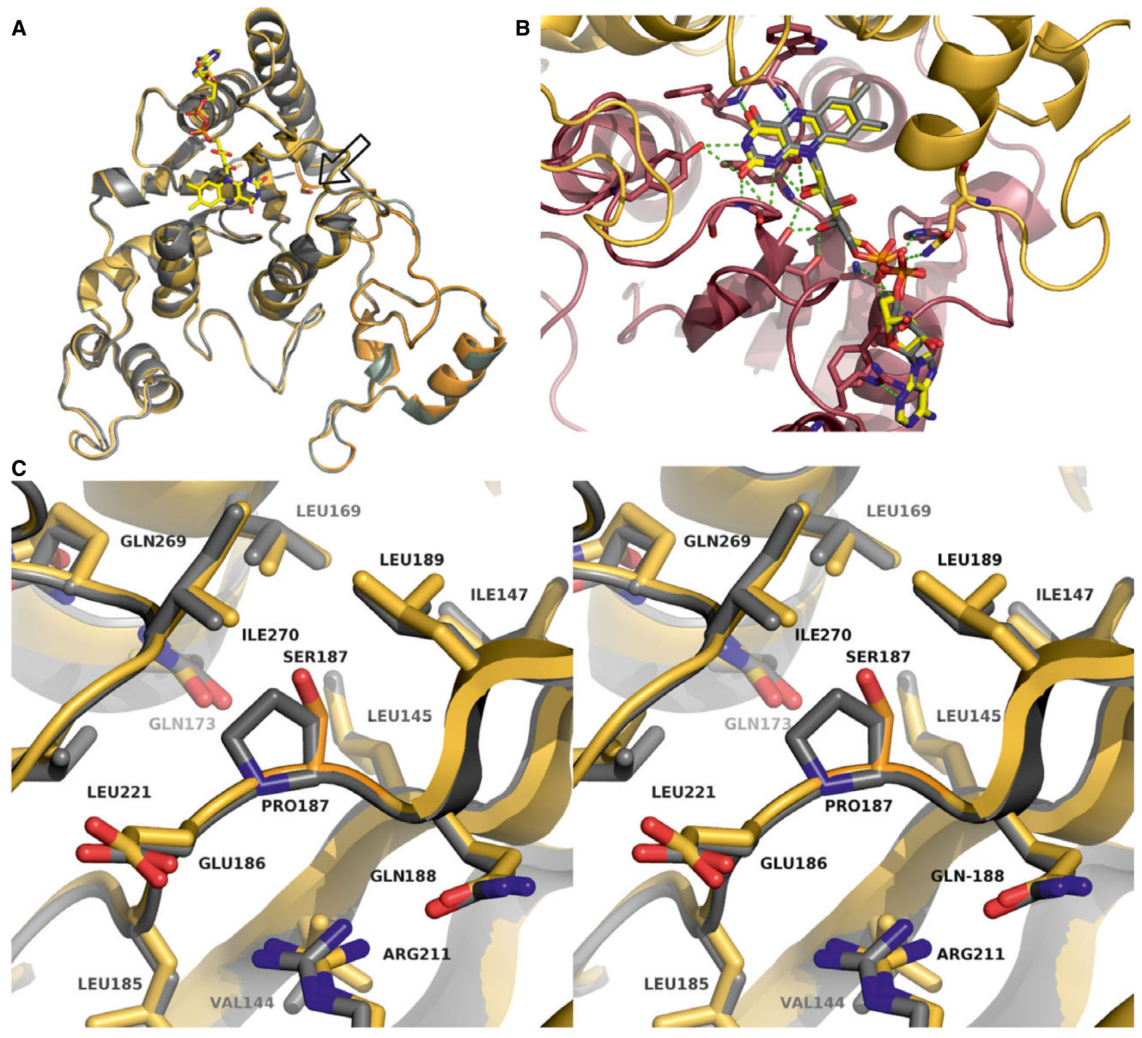
The crystal structures of NQO1 P187S and NQO1 are almost identical. (A) Crystal structure of an NQO1 P187S (gold) subunit superimposed on NQO1 (gray) (PDB: 1d4a); the amino acid exchange site is represented as a stick model (located on the right side of the FAD cofactor isoalloxazine ring). (B) Close-up view of the FAD-binding site in NQO1 P187S. The FAD molecule is represented as sticks model, and the two subunits of NQO1 are represented as cartoons and shown in red and gold. Hydrogen bonds between FAD and the protein backbone are depicted as green dashed lines. (C) Stereo representation showing the site of the amino acid replacement; NQO1 P187S (gold) is superimposed on NQO1 (gray).

The observed qualitative and quantitative differences in the physical properties of FAD binding and catalysis suggested structural perturbations in the FAD-binding pocket of NQO1 P187S. To analyze these putative structural differences, we solved the X-ray crystal structure of NQO1 P187S (Fig. 2B) (for data collection and refinement statistics, see Table 2). Initial crystallization attempts were successful in the presence of an equimolar concentration of Cibacron blue and excess dicoumarol. These crystals diffracted to 2.7 Å and showed a structural topology (Fig. 3A) that was almost identical to that of the NQO1 crystal structure [15,16], with an rmsd of 0.33 Å for 249 Cα atoms. The FAD-binding site of NQO1 P187S showed no discernible differences, with both the flavin isoalloxazine ring and the AMP moiety engaging in the same interactions as in NQO1 (Fig. 3B). The same was true for the region around the amino acid exchange site, where no significant structural differences between NQO1 and NQO1 P187S were observed (Fig. 3C). Although our spectroscopic and kinetic measurements have documented the effects of the proline to serine exchange on catalytic function, the determined crystal structure of NQO1 P187S is almost identical to NQO1, and hence does not provide a structure-based explanation for the lower enzymatic activity. However, we noticed that crystallization of NQO1 P187S was much slower than that of NQO1 (weeks rather than days), suggesting that NQO1 P187S might be present in a less ordered state in solution. This prompted us to employ NMR spectroscopy to characterize the structural properties of NQO1 and NQO1 P187S in solution.

**Fig. 4:**
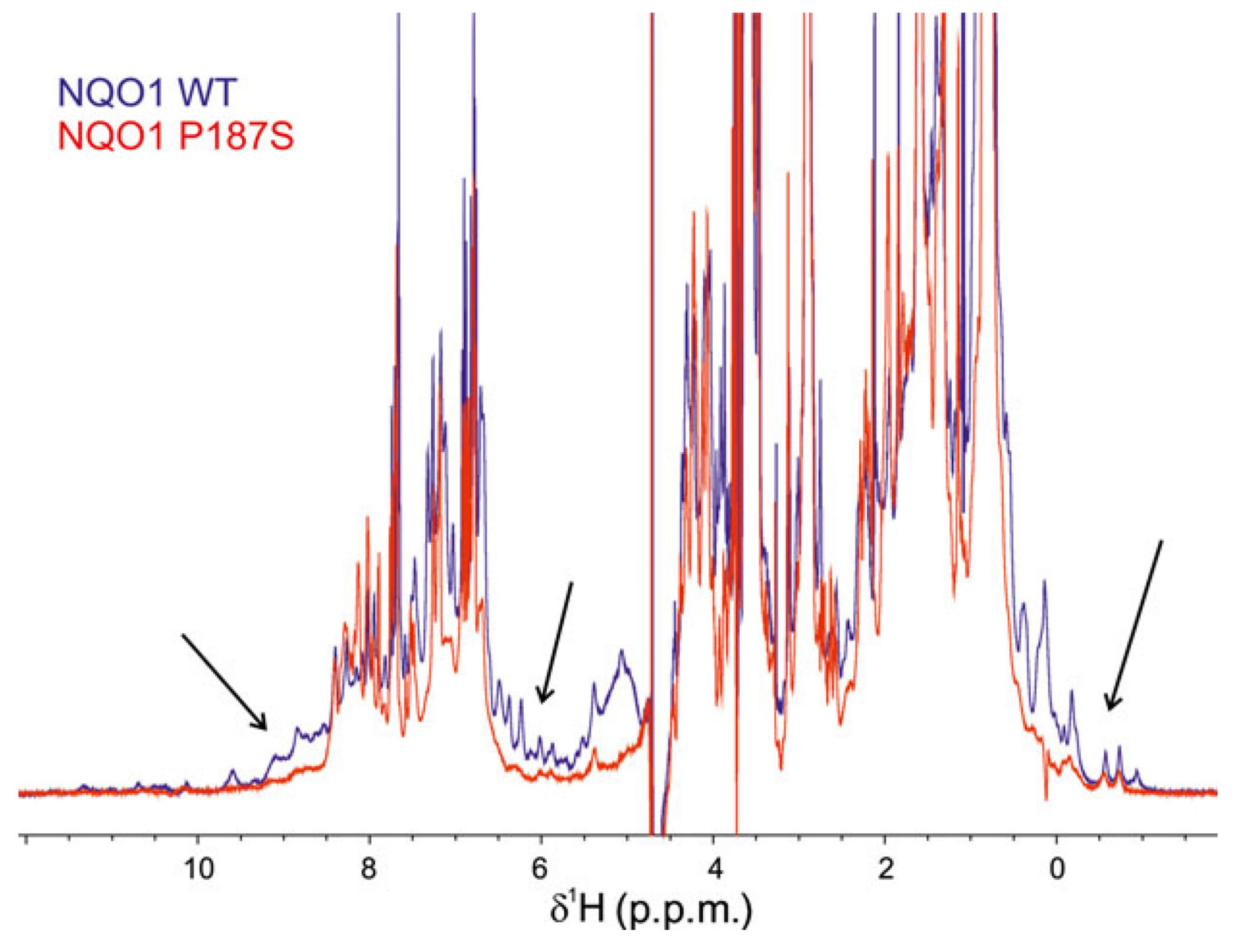
NQO1 P187S shows different structural topology in solution to that of NQO1. Intensities of ^1^H-NMR spectra for NQO1 (blue) and NQO1 P187S (red) were normalized on the methyl region (0.9 ppm) and superimposed. Signals outside the random coil regions (arrows), which reflect tertiary structure, are much more pronounced for NQO1 than for NQO1 P187S, indicating its loss of structure in solution.

**Fig. 5:**
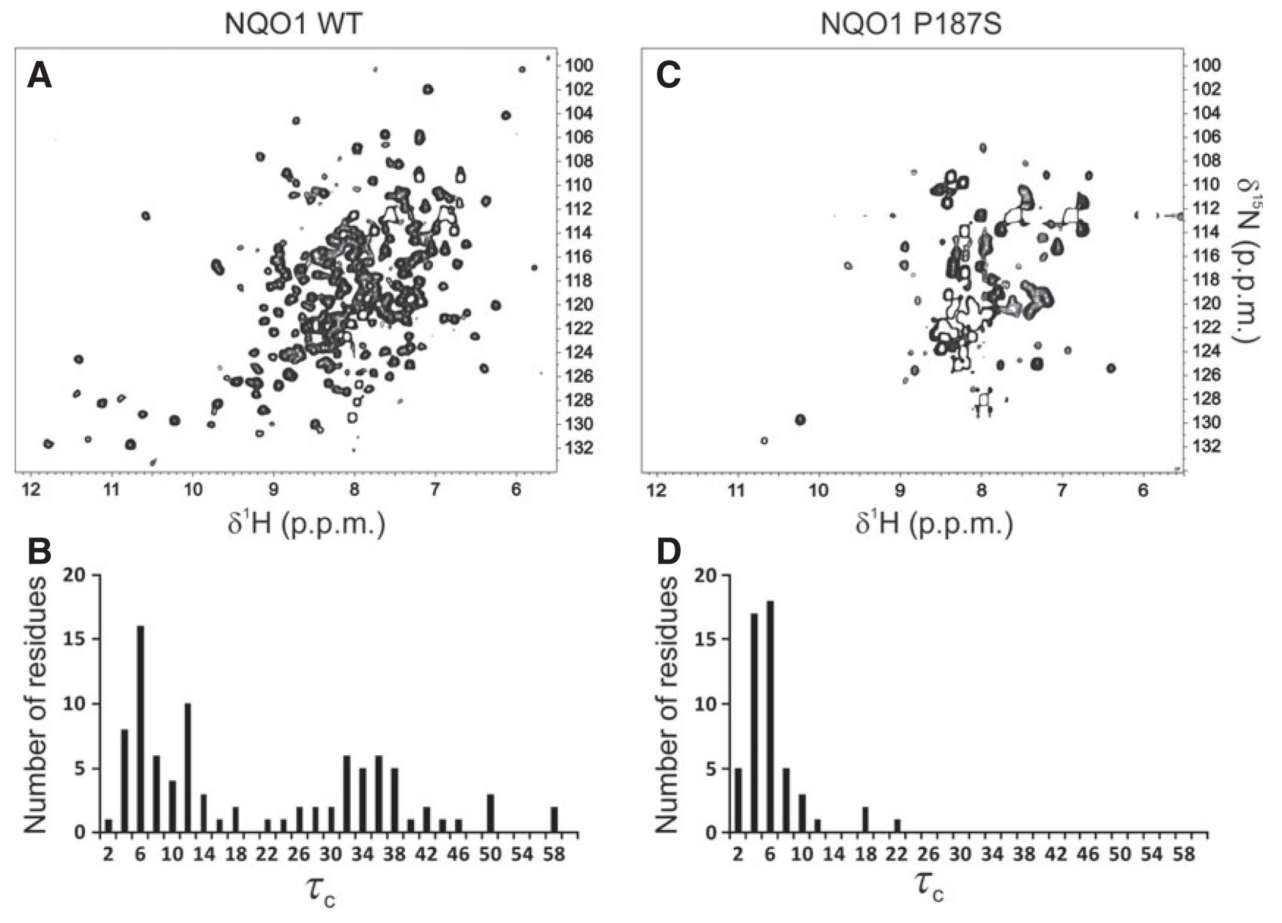
Destabilization of the NQO1 P187S structure in solution. Two-dimensional ^1^H/^15^N-HSQC spectra of NQO1 and NQO1 P187S are shown in (A) and (C), respectively. The distribution of rotational correlation times (τ_c_ in ns) for NQO1 and NQO1 P187S are shown in (B) and (D), respectively. The almost complete absence of well-dispersed signals in the HSQC spectrum and of high τ_c_ values are indicative of the increased mobility in NQO1 P187S.

**Fig. 6:**
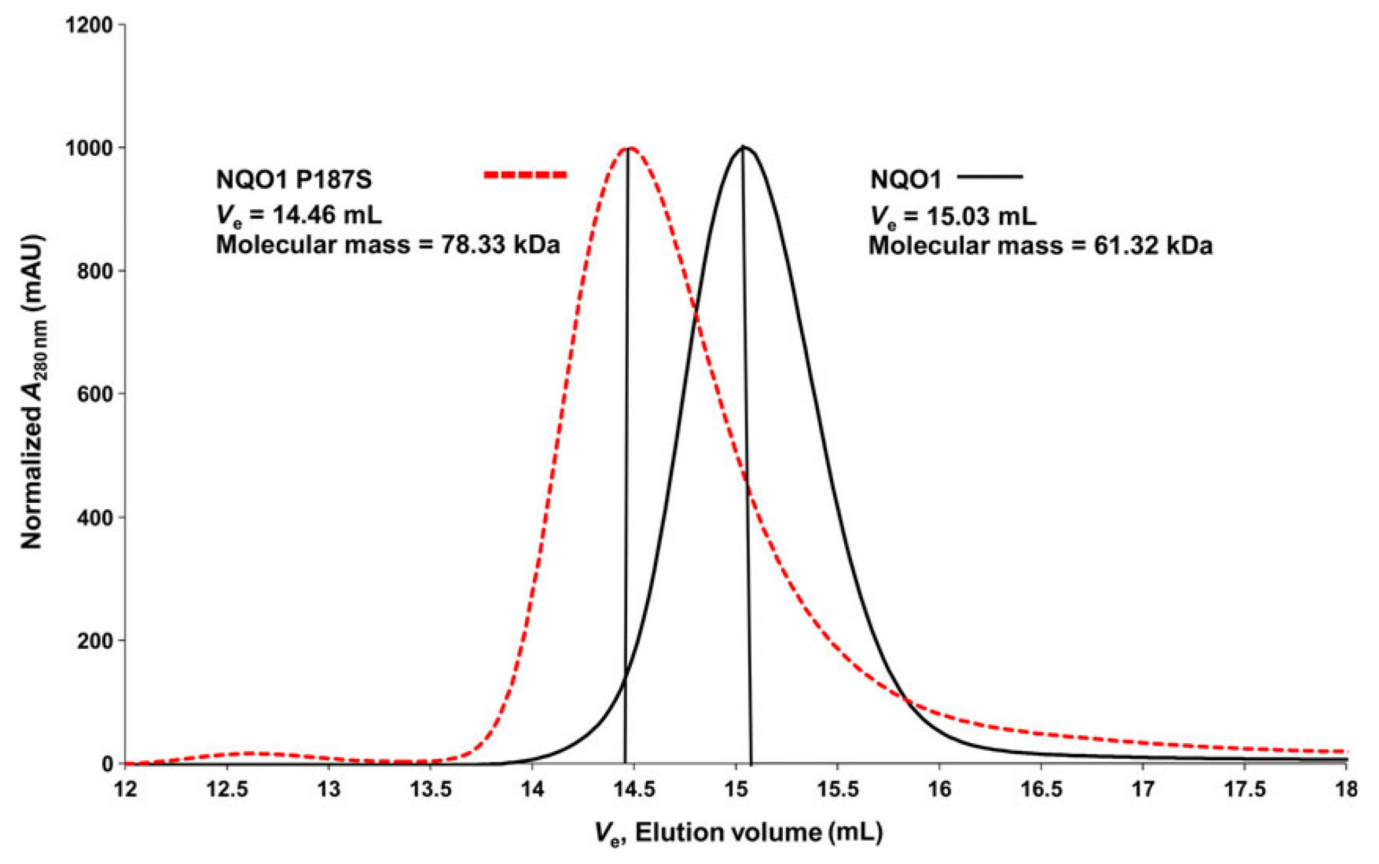
Analytical size exclusion chromatography of NQO1 and NQO1 P187S. Analytical size exclusion chromatograms of purified NQO1 (continuous line) and NQO1 P187S (dashed line) obtained with a Superdex 200 (10/300) analytical column are shown. The calculated molecular masses of NQO1 (61.3 kDa) and NQO1 P187S (78.33 kDa) indicate that both proteins exist in dimeric forms. The lower elution volume observed for NQO1 P187S indicates deviation from the globular structure adopted by NQO1, thus supporting our NMR data showing that NQO1 P187S has a more disordered structure than NQO1.

Both 1D proton spectra (Fig. 4) and 2D ^1^H/^15^N-HSQC measurements (Fig. 5A,C) obtained with uniformly ^15^N-labeled proteins revealed significant structural differences between NQO1 and NQO1 P187S. Signals indicative of a well-folded protein (e.g. peaks below 0 ppm in the ^1^H-NMR spectrum) and well-dispersed signals beyond ~ 8.5 ppm in ^1^H/^15^N-HSQC spectra were significantly more pronounced for NQO1 than for NQO1 P187S (Fig. 4). To gain further insights into the dynamic behavior, we carried out ^15^N relaxation measurements. Histograms showing the distribution of the rotational correlation time are presented in Fig. 5B,D. Short correlation times (< 20 ns) are indicative of highly flexible residues, whereas the well-structured regions of a protein in the molecular mass range of NQO1 should give rise to values of ~ 30–40 ns. In the case of NQO1 P187S, more signals with very short correlation times were observed. For example, 50 peaks with a rotational correlation time below 10 ns were found for NQO1 P187S, and only 32 for NQO1, indicating that, overall, the very flexible regions of NQO1 P187S encompass a larger proportion than in NQO1. On the other hand, much longer correlation times of up to 58 ns were observed for NQO1 (compare Fig. 5B and Fig. 5D). Peaks missing in the ^1^H/^15^N-HSQC spectrum of NQO1 P187S (Fig. 5C) are probably in the intermediate–fast motional regime (*k*_ex_ ~ ms), leaving only the very flexible residues observable. In order to confirm that the reduced number of signals in the ^1^H/^15^N-HSQC spectrum of NQO1 P187S (Fig. 5C) was not attributable to the formation of protein aggregates, we performed analytical size exclusion chromatography (Fig. 6). The calculated size of NQO1 (61 kDa) agrees well with the theoretical size of NQO1 dimers (62 kDa); on the other hand, the calculated size of NQO1 P187S (78.33 kDa) suggests that the hydrodynamic radius is larger than that of NQO1. In addition, dynamic light scattering experiments failed to detect protein aggregation even with extended periods of time (up to 8 h). Thus, size exclusion chromatography and dynamic light scattering data concur with the NMR data that NQO1 P187S has a larger disordered portion than is present in the globular structure of NQO1. As signals indicative of a native structure are present in both 1D (Fig. 4) and 2D (Fig. 5A,C) spectra, but with significantly reduced intensity, we assume that, in addition to there being more extended highly mobile regions in NQO1 P187S, the percentage of fully structured protein is lower, in line with the substantially slower crystallization of the variant. Overall, contrasting structural evidence from X-ray crystallography and NMR spectroscopy led us to assume that both disordered and structured conformations of NQO1 P187S exist simultaneously. However, it is apparent that the percentage of NQO1 P187S showing a native conformation is significantly reduced, as rapid reaction experiments (Table 1) showed that the catalytic activity of NQO1 P187S is severely compromised.

**Fig. 7:**
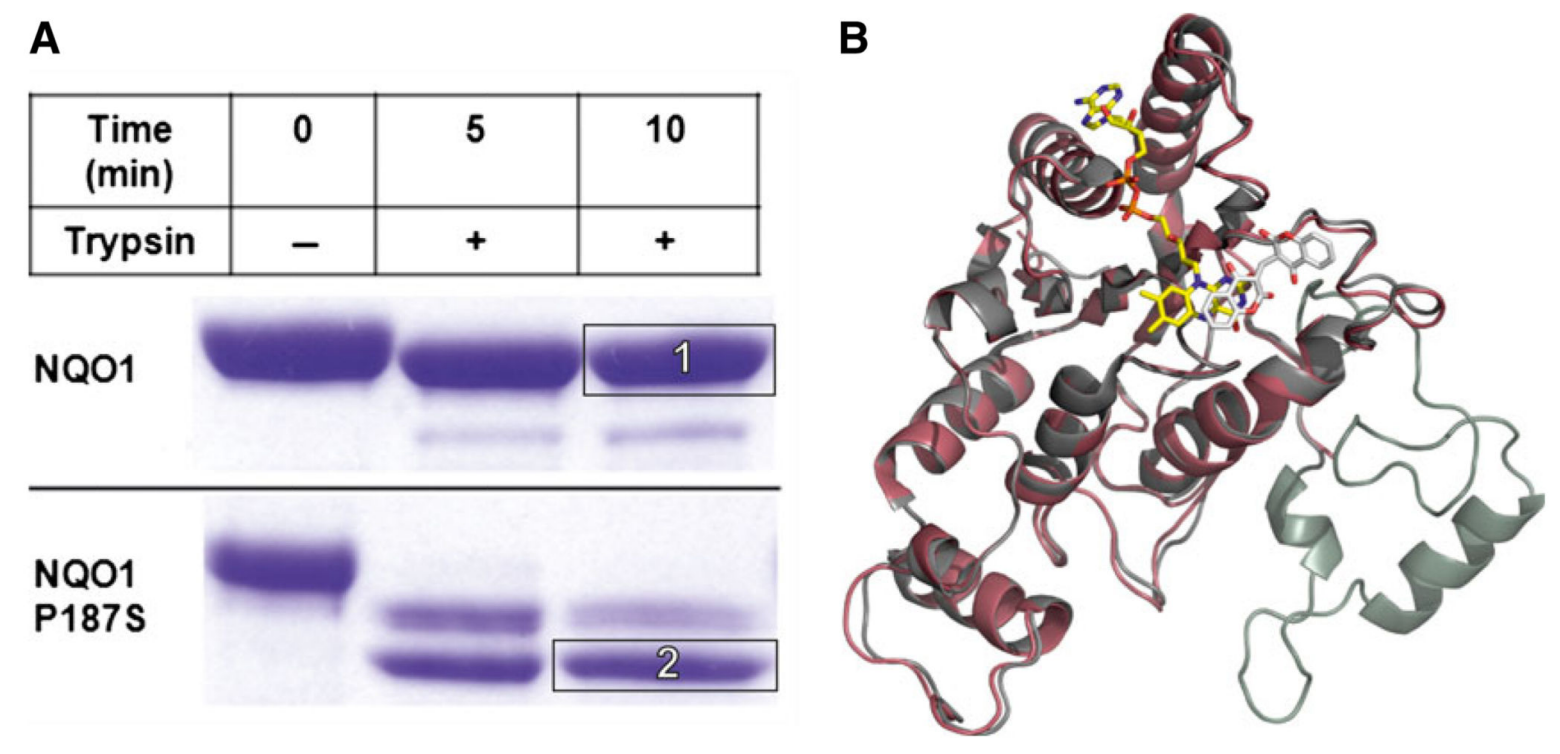
The C-terminus of NQO1 P187S is flexible. (A) Limited proteolysis of NQO1 and NQO1 P187S by trypsin analyzed with SDS/PAGE. The C-terminus of NQO1 P187S was proteolysed within 5 min, indicating that it is flexible. (B) Superimposed X-ray crystal structures of NQO1 (gray) and NQO1 P187S (C-terminal truncation) (magenta). The C-terminus of NQO1 P187S was cleaved by an unknown protease during the crystallization procedure, confirming our hypothesis that the C-terminus is flexible.

**Fig. 8:**
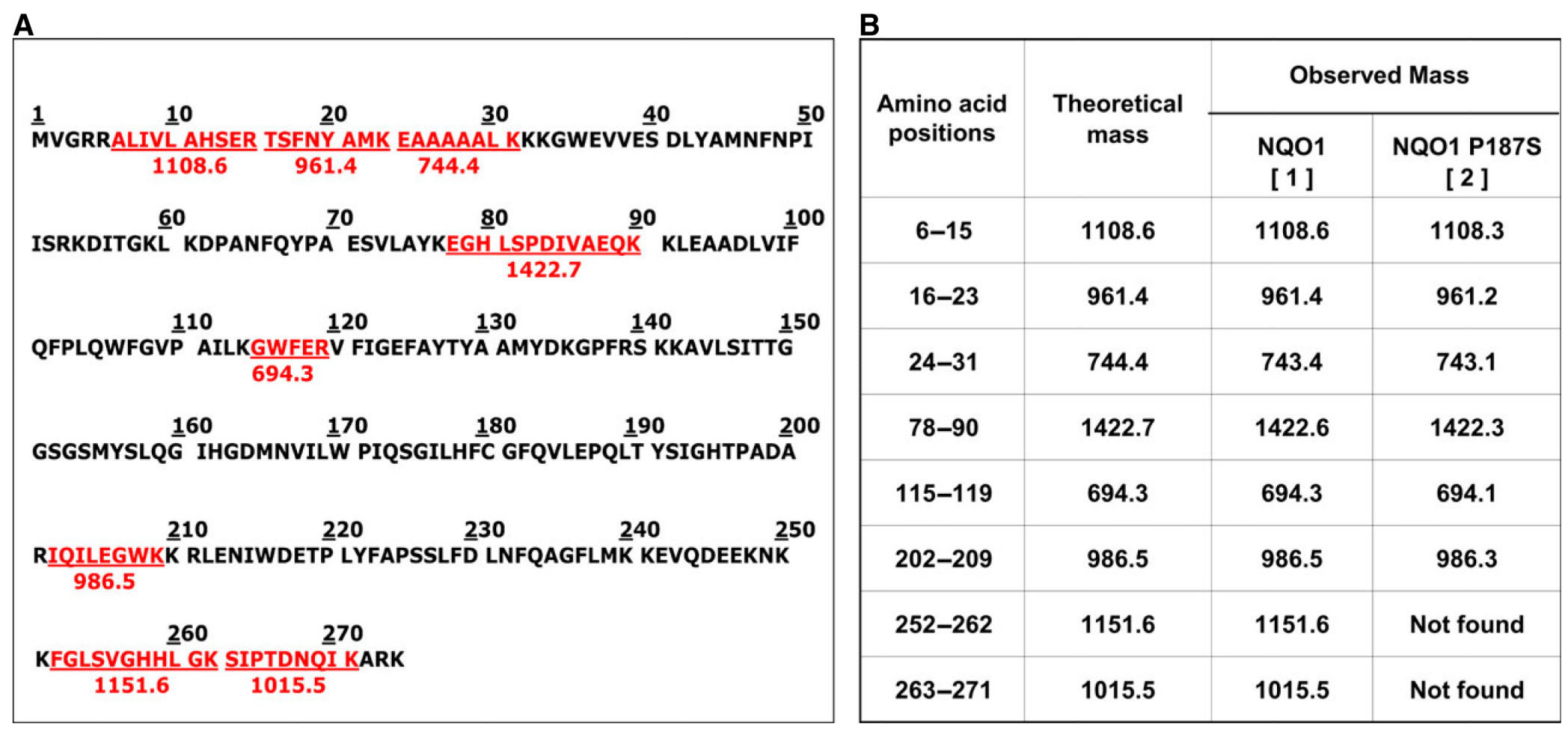
MALDI-TOF MS analysis of NQO1 proteins after limited proteolysis. The C-terminus of NQO1 P187S is more rapidly proteolysed than that of NQO1. (A) Theoretical tryptic digest of NQO1 generated by the expasy peptidecutter tool; peptide sequences colored in red were used as a peptide fingerprint for analysis of the MALDI-TOF MS data. (B) Table listing the peptides from SDS/PAGE bands after MALDI-TOF MS of NQO1 and NQO1 P187S (insets 1 and 2 in Fig. 7A).

In a different batch of crystallization trials, crystals were obtained for NQO1 P187S in the absence of Cibacron blue; these diffracted to 2.2 Å (Fig. 2C) (for data collection and refinement statistics, see Table 2). With these crystals, electron density was not obtained for the last 50 C-terminal amino acids (Fig. 7B). Cibacron blue and dicoumarol are both competitive inhibitors of NADH [17,18], indicating that occupation of the NADH-binding pocket stabilizes the C-terminus and thus prevents proteolysis during crystallization. Apart from the missing C-terminus, this crystal structure showed no difference from NQO1 in the overall topology of the structure or the active site. ESI-MS analysis of a protein crystal taken from the same crystallization drop confirmed that the protein was truncated, indicating that the C-terminus was lost because of proteolytic cleavage. Closer inspection of the missing C-terminal amino acids revealed that the cleavage of NQO1 P187S had occurred at amino acid 224. To gain further insights into the loss of the C-terminus during the crystallization procedure, we performed limited proteolysis experiments (Fig. 7A). NQO1 P187S was rapidly cleaved by trypsin, whereas NQO1 was less susceptible to tryptic cleavage. Complete in-gel tryptic digestion of the protein bands (insets 1 and 2 in Fig. 7A) and subsequent peptide mass fingerprinting by MALDI-TOF MS confirmed that the C-terminus was cleaved within 5 min in NQO1 P187S but was still present in NQO1 (Fig. 8). The C-terminus of NQO1 was cleaved much more slowly than in NQO1 P187S; that is, more than half of the protein retained the C-terminus even after 160 min of incubation. To assess the role of the C-terminus in catalysis, we generated C-terminally truncated proteins (NQO1 Δ50 and NQO1 P187S Δ50) and determined the rates of reduction. In the case of NQO1 P187S Δ50, the bimolecular rate constants for NADH and NADPH were only marginally affected by the C-terminal truncation (Table 1). In contrast, the bimolecular rate constants for NQO1 Δ50 (with either NADH or NADPH as reducing agent) were decreased 1000-fold compared with that of NQO1 (Table 1), proving that the C-terminus is important for enzymatic activity. Thus the P187S amino acid exchange affects the kinetics of FAD reduction to a similar extent as the complete removal of the C-terminus in NQO1 Δ50. This finding supports our conclusion that the C-terminus is not properly associated with the core domain in NQO1 P187S. This perturbation of the interdomain contact leads to a higher degree of flexibility of the C-terminus, compromising the catalytic efficiency of the variant.

Overall, our structural and biochemical experiments reveal that the proline to serine replacement disrupts the interaction of the core with the C-terminus, resulting in a disordered tertiary structure that ultimately leads to greatly diminished enzymatic activity. A plausible explanation for the disruption of the interaction between the core and the C-terminus in NQO1 P187S is the replacement of the hydrophobic proline with a hydrophilic serine, leading to unfavorable contacts with hydrophobic residues surrounding position 187 (Leu145, Ile147, Leu169, Leu189, and Ile170; Fig. 2C), and thus destabilizing the contact area between the C-terminus and the hydrophobic core domain.

**Fig. 9:**
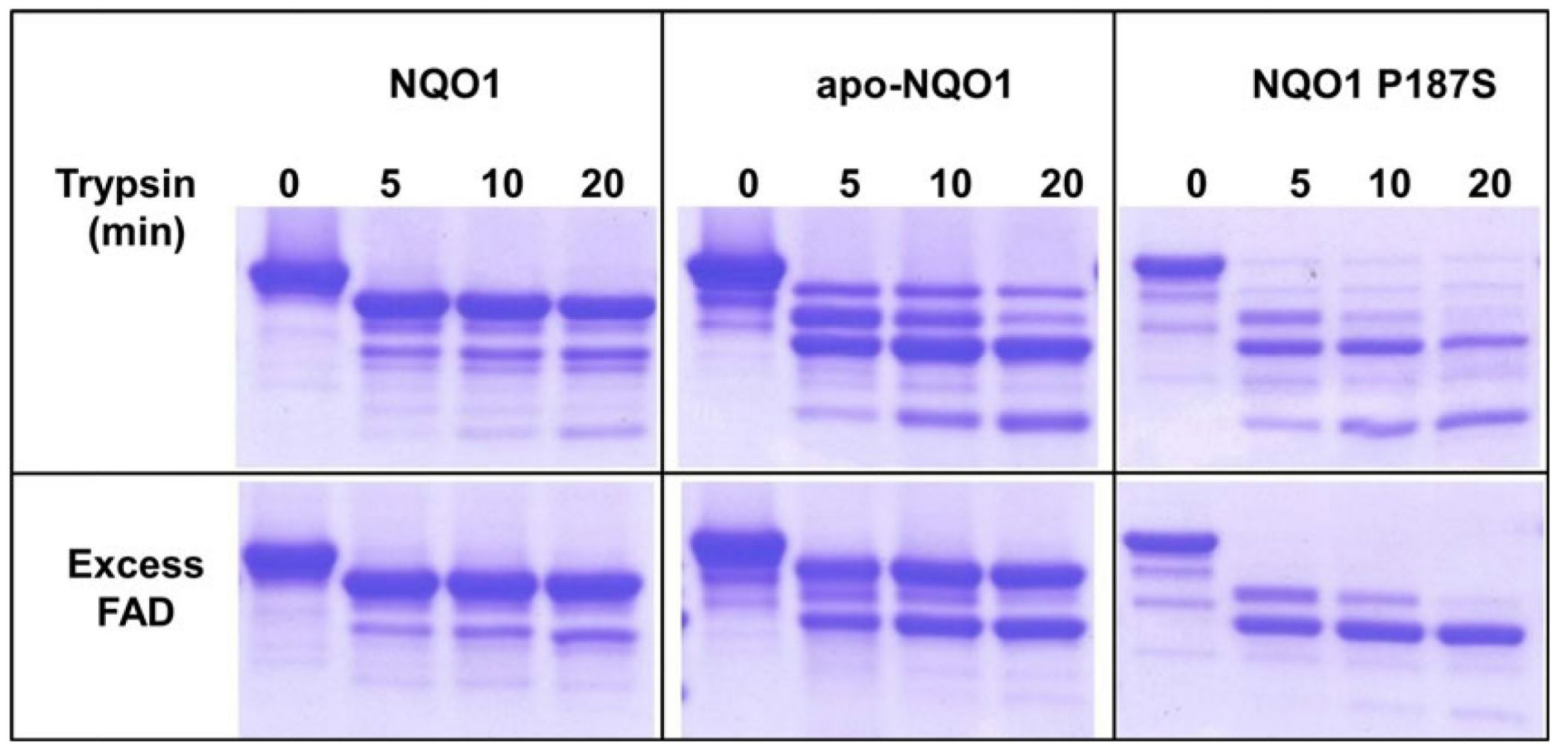
Interdomain association between the C-terminus and core domain of NQO1 P187S is not stabilized by the presence of FAD. NQO1, apo-NQO1 and NQO1 P187S were subjected to limited tryptic digestion, in the presence (bottom panel) or absence (top panels) of excess FAD. Protein solution (30 μm) was digested with 2 ng·μL^−1^ sequencing-grade modified trypsin in the presence or absence of 300 μm FAD, and analyzed with SDS-PAGE. In the presence of FAD, apo-NQO1 was stabilized, whereas the C-terminus of NQO1 P187S was completely cleaved within 5 min. NQO1 P187S was stabilized after proteolysis of the C-terminus, indicating that FAD does not stabilize the interaction between the C-terminus and the core domain. However, the core domain appears to be more stable in the presence of FAD in NQO1 P187S.

The evidence presented so far in this article provides a rationale for the decrease in enzymatic activity of a crucial metabolic enzyme NQO1 with a point mutation. However, information is scarce as to how the single amino acid exchange affects the protein interaction properties of NQO1 P187S. NQO1 physically interacts with the tumor suppressor proteins p53 and p73 in an NADH-dependent manner, and protects them from 20S proteasomal degradation, whereas NQO1 P187S is unable to prevent the degradation of p53 and p73 [3,14]. Moscovitz *et al.* showed that supplementing NQO1*2 cell lines with increasing concentrations of riboflavin, the precursor of FAD, stabilizes the intracellular levels of NQO1 P187S [14]. The authors of this study proposed that riboflavin or FAD analogs may offer a potential therapeutic avenue for NQO1*2 homozygous individuals, who are sensitive to benzene hematotoxicity and are at increased risk of developing various cancers. In order to determine whether the evidence provided by the authors also holds true *in vitro*, we repeated the limited proteolysis experiments (Fig. 7A) in the presence of a 10-fold molar excess of FAD (Fig. S9). In contrast to the *in vivo* results, addition of FAD to NQO1 P187S did not prevent the cleavage of the C-terminus by trypsin (Fig. 9), suggesting that the intracellular levels of this protein variant might not be dependent on FAD levels.

The overexpression of NQO1 in various solid tumors and its ability to activate quinone-based chemotherapeutics have made it a subject of numerous investigations, leading to proposals that NQO1 is an attractive target for anticancer treatment [6]. The ability of NQO1 to bioactivate quinone pharmacophores to cytotoxic two-electron-reduced hydroquinones is of paramount importance for the proposed anticancer treatment. However, it has always been a matter of debate as to whether it is a single-electron reductase, such as NADPH:cytochrome P450 reductase, or a two-electron reductase, such as NQO1, which bioactivates quinone pharmacophores. A recent study reported the association of the NQO1*2 homozygous genotype with adverse breast cancer outcomes and poor survival rates after anthracycline-based chemotherapy (17% for NQO1*2 versus 75% for other genotypes) [10]. The authors proposed that NQO1 influences the outcome of epirubicin treatment through at least three mechanisms: the p53 pathway; the tumor necrosis factor–nuclear factor-κB pathway; and direct detoxification of reactive oxygen species. The wealth of information available on the involvement of the NQO1*2/NQO1 P187S homozygous genotype/variant in cancer development and the failure to activate chemotherapeutics stands in contrast to the lack of knowledge on the loss of function at the molecular and structural levels. The results of our study further underline the importance of the *NQO1* genotype in cancer patients, and indicate that NQO1*2 homozygous patients will respond poorly to quinone-based chemotherapy [9,10]. Equally, the quinone-based drug EPI-743, which specifically targets NQO1, was granted an orphan designation for treating mitochondrial diseases. However, no information is currently available on how patients with the homozygous NQO1*2 genotype respond to this drug [19].

In conclusion, our study provides sufficient biochemical, structural and spectroscopic evidence to rationalize the loss of function in the common human NQO1 P187S variant. In addition, the results of our study explain the resistance to treatment with anthracycline-based cancer chemotherapeutics, e.g. epirubicin. Equally, our study emphasizes the importance of combining structural methods to enhance our understanding of the effects that SNPs have on protein properties, in particular folding dynamics. On the basis of our results, we suggest that it is greatly beneficial to analyze *NQO1* allelic status prior to treatment with quinone-based chemotherapeutics. In the case of NQO1, it is conceivable that efforts to reinforce interdomain contacts, e.g. with small-molecular chaperones, may lead to repopulation of the catalytically competent native structure, thus paving the way for more efficient treatment with quinone-based anticancer drugs.

## Experimental procedures

### Molecular cloning of *NQO1*

The *NQO1* gene sequence (UniProtKB/Swiss-Prot: P15559) was codon-optimized for *Escherichia coli* expression and chemically synthesized (GeneArt, Carlsbad, CA, USA). The *NQO1* genes were cloned into the pET28a (Merck, Darmstadt, Germany) vector with *Nde*1 and *Xho*1 restriction sites to encode for an N-terminal histidine-tagged fusion protein by the use of gene-specific primers (Eurofins, Luxembourg). *NQO1 P187S, NQO1 Δ50* and *NQO1 P187S Δ50* were generated with the Quik-Change II XL Site-Directed Mutagenesis Kit (Santa Clara, CA, USA).

### Protein expression and purification

Protein expression was carried out in LB broth (5 g·L^−1^ sodium chloride) containing 50 μg·L^−1^ kanamycin. Fresh medium was inoculated with 40 mL·L^−1^ of an overnight culture and grown to a *D*_600 nm_ of 0.95 before induction with 0.4 mm isopropyl thio-β-d-galactoside. Cells were harvested at 17 600 ***g*** for 5 min, resuspended in 1% saline solution, and pelleted at 3700 ***g*** at 4 °C for 45 min. Cell pellets were resuspended in lysis buffer (50 mm Hepes, 150 mm NaCl, 10 mm imidazole, pH 7.0) with 2 mL of buffer per 1 g of pellet. One milligram of FAD (disodium salt hydrate; Sigma Aldrich, St Louis, MO, USA) and 10 μL of protease inhibitor cocktail for use in the purification of histidine-tagged proteins in dimethylsulfoxide solution (Sigma Aldrich, St Louis, MO, USA) were added per 25 mL of slurry. Cell disruption was achieved by sonication with a Labsonic P instrument (Sartorius, Göttingen, Germany) with 70% intensity and 0.5 pulse for 10 min on ice. The cell lysate was centrifuged at 38 759 ***g*** for 30 min, and the supernatant was loaded onto a 5-mL HisTrap FF (GE Healthcare, Little Chalfont, UK) column previously equilibrated with 50 mm Hepes, 150 mm NaCl, and 20 mm imidazole (pH 7.0). The column was washed with 50 mL of 50 mm Hepes, 150 mm NaCl, and 50 mm imidazole (pH 7.0), after which proteins were eluted with 50 mm Hepes, 150 mm NaCl, and 300 mm imidazole (pH 7.0). Fractions containing target proteins were pooled and concentrated with centrifugal filter units (Amicon Ultra-15, 30 k; Millipore, Billerica, MA, USA). Concentrated protein was applied to a HiLoad 16/60 Superdex 200 prep grade column (GE Healthcare, Little Chalfont, UK) equilibrated with 50 mm sodium phosphate and 150 mm NaCl (pH 7.0) for further purification. The fractions containing target proteins were collected, and this was followed by rebuffering with a PD-10 desalting column (GE Healthcare, Little Chalfont, UK) in 50 mm Hepes (pH 7.0). The protein solutions were shock frozen and stored at −80 °C if not used immediately.

### Apoprotein preparation

Apoproteins were prepared according to the method described by Hefti *et al.* [20]; any exceptions to the protocol are mentioned below. NQO1 or NQO1 P187S was applied to a 5-mL HisTrap FF (GE Healthcare, Little Chalfont, UK) column previously equilibrated with 50 mm Hepes, 150 mm NaCl, and 20 mm imidazole (pH 7.0). The column was washed with 50 mL of buffer containing 50 mm Hepes, 150 mm NaCl, 2 m urea, and 2 m KBr (pH 7.0), and then re-equilibrated with 50 mm Hepes, 150 mm NaCl, and 20 mm imidazole (pH 7.0) (~ 25 mL). Apo-protein was then eluted with 50 mm Hepes, 150 mm NaCl, and 300 mm imidazole (pH 7.0). Removal of FAD was verified spectrophotometrically after rebuffering of the protein into 50 mm Hepes (pH 7.0), by the use of PD-10 desalting columns (GE Healthcare, Little Chalfont, UK).

### UV–visible absorption difference titration

Difference titrations were carried out in tandem cuvettes (two separated chambers in the sample and reference cuvettes) with a Specord 200 plus spectrophotometer (Analytik Jena, Jena, Germany) at 25 °C. The cuvettes were filled with 800 μL of 50 μm apo-protein solution in buffer (50 mm Tris, 150 mm NaCl, pH 7.5) in one chamber, and 800 μL of buffer in the other chamber. The titration experiment was performed by addition of FAD (1 mm stock solution) to the apo-protein in the sample cuvette and to the buffer in the reference cuvette. The same volume of buffer was added to the apo-protein solution in the reference cell in order to obtain the same protein concentrations in the sample and reference cuvette. After completion of the additions and careful mixing with a Pasteur pipette (one for each chamber being used throughout the titration experiment), an absorption spectrum was recorded (250–600 nm).

### ITC

A VP-ITC system (MicroCal; GE Healthcare, Little Chalfont, UK) was used for calorimetric determination of the dissociation constants for FAD. All experiments were performed at 25 °C in 50 mm Hepes (pH 7.0), and solutions were degassed before measurements. The titration experiments were performed with (290 ± 10) μM apo-protein solution in the syringe and (27 ± 1) μm FAD solution in the measurement cell. The concentrations of FAD and the apo-protein were determined spectrophotometrically. All experiments comprised 35 injections (initial injection of 2 μL, with an injection duration time of 4 s, and a spacing time of 300 s, followed by 34 injections of 6 μL, with an injection duration time of 5.4 s, and a spacing time of 300 s). Standard measurements without FAD were subtracted and the first measurement point was rejected; the remaining data points were analyzed on the assumption of a single-site binding model with origin version 7.0 (Micro-Cal) for ITC data analysis.

### Rapid reaction studies

Stopped-flow measurements were carried out with a Hi-Tech (SF-61DX2) stopped-flow device (TgK Scientific Limited, Bradford-on-Avon, UK), positioned in a glove box from Belle Technology (Weymouth, UK), at 4 °C. Buffers were flushed with nitrogen, and this was followed by incubation in the glove box environment. The enzyme and substrate solutions were deoxygenated by incubation in the glove box environment, and then diluted with buffer to the required concentrations. Enzyme and substrate were rapidly mixed in the stopped-flow device, and FAD oxidation and reduction were measured by monitoring changes at *A*_455 nm_ with a KinetaScanT diode array detector (MG-6560; TgK Scientific Limited). Initial rates were calculated by fitting the curves with a two-exponential function.

The reductive half-reaction was investigated by mixing proteins (40 μm) in 50 mm Hepes (pH 7.0) with NADPH or NADH in the stopped-flow device. In the case of NQO1, the NAD(P)H concentrations were in the range of 50–200 μm, and in the case of NQO1 P187S, NQO1 Δ50 and NQO1 P187S Δ50, the NAD(P)H concentrations were between 50 μm and 5 mm. The absorption decrease was monitored at 455 nm. To study the oxidative half-reaction, proteins (40 μm) in 50 mm Hepes (pH 7.0) were first reduced by the addition of equimolar amounts of NADH. The reduced enzymes were then mixed with either benzoquinone or menadione (30–100 μm), and the reoxidation of the reduced FAD cofactor was monitored at 455 nm.

### Crystallization conditions used for obtaining the NQO1 P187S structure

NQO1 P187S at 11 mg·mL^−1^ in 50 mm Hepes (pH 7.0) saturated with dicoumarol and equimolar Cibacron blue was crystallized by the microbatch method in a precipitating solution containing 60% Tacsimate (pH 7.0) (Hampton Research Index Screen, condition 29), and incubated at 289 K. The total drop volume was 1 μL, with equal amounts of protein and precipitant solution. The bluish crystal grew to full size (~ 85 μm) within 30 days. Crystals were harvested from their mother liquor with CryoLoops (Hampton Research), and flash-cooled in liquid nitrogen.

### Crystallization conditions used for obtaining the NQO1 P187S structure (C-terminal truncation)

NQO1 P187S at 11 mg·mL^−1^ in 50 mm Hepes (pH 7.0) saturated with dicoumarol was crystallized by the vapour batch method in a precipitating solution containing 5 mm cobalt(II) chloride hexahydrate, 5 mm nickel(II) chloride hexahydrate, 5 mm cadmium chloride hydrate, 5 mm magnesium chloride hexahydrate, 100 mm Hepes (pH 7.5), and 12% (w/v) poly(ethylene glycol) 3350 (Hampton Research Index Screen, condition 64), and incubated at 289 K. The total drop volume was 1 μL, with equal amounts of protein and precipitant solution. The yellow crystals grew to full size (~ 80 μm) within 2 months. Crystals were harvested from their mother liquor with CryoLoops (Hampton Research), and flash-cooled in liquid nitrogen.

### Structure determination and refinement of NQO1 P187S

A complete dataset was collected from a single crystal at beamline ID-29 (λ = 1.0 Å) at the European Synchrotron Radiation Facility. The dataset was collected to 2.69-Å resolution from an orthorhombic crystal (space group *I*222). Data were processed with xds [21]. Analysis of the dataset with phenix [22] showed a pseudomerohedral twin operator with a twin law ‘−k, −h, −l’ and a twin fraction of 39% according to Yeates’ *L*-test [23]. Patterson analysis revealed a significant off-origin peak of 49.5% of the origin peak, indicating pseudotranslational symmetry. The calculated Matthews coefficient [24] indicated the presence of two molecules per asymmetric unit. The structure was solved by molecular replacement with phaser [25], and the detwinned dataset was produced with detwin [3]. The partially refined structure of NQO1 P187S (C-terminal truncation) was used as search template. The best phaser result (based on log-likelihood statistics) was further used as the input model for the automated chain-tracing/rebuilding program buccaneer [26]. *R*_free_ values were computed from 5% randomly chosen reflections that were not used during refinement [27] Structure refinement and model rebuilding were carried out with phenix [22] and coot [28,29] by alternating real-space fitting against σ_A_-weighted 2*F*_o_ − *F*_c_ and *F*_o_ − *F*_c_ electron density maps and least-square optimizations. Seven water molecules were manually placed into strong peaks of the difference electron density map. The final model was refined to *R* = 18.5% and *R*_free_ = 20.7%. Details of the data reduction and structure refinement are shown in Table 2.

Electron density could not be observed for residues 1–3 and residue 274 in one protomer, and for residues 1–3 in the other protomer. The cofactor FAD and the ligand Cibacron blue were placed manually into the difference electron density map. Electron density for dicoumarol could not be identified. Validation of the structure was carried out with molprobity [30], yielding a Ramachandran plot with 93.5% of the residues in favored regions, 6.5% in allowed regions, and none in disallowed regions. Prediction of the biologically active form of NQO1 P187S was performed with the pisa server [31]. Figures were created with pymol [32].

### Structure determination and refinement of NQO1 P187S (C-terminal truncation)

A complete dataset for NQO1 P187S with a C-terminal truncation was collected from a single crystal at beam-line XRD1 (λ = 1.1 Å) at ELETTRA (Trieste, Italy). The dataset was collected to 2.2-Å resolution from a tetragonal crystal (space group *P*4_1_2_1_2). The data were processed with xia2 [33]. The calculated Matthews coefficient [24] indicated the presence of one molecule per asymmetric unit. The structure was solved by molecular replacement with phenix automr [22] and phaser [25], with the structure of the NQO1 protomer [Protein Data Bank (PDB) entry 1qbg] as the search template. The best phaser result (based on log-likelihood statistics) was further used as the input model for the automated chain-tracing/rebuilding program buccaneer [26]. *R*_free_ values were computed from 5% randomly chosen reflections that were not used during refinement [27]. Structure refinement and model rebuilding were carried out with phenix [22] and coot [28,29] by alternating real-space fitting against σ_A_-weighted 2*F*_o_ − *F*_c_ and *F*_o_ − *F*_c_ electron density maps and least-square optimizations. Water molecules were placed into the difference electron density map, and accepted or rejected according to geometry criteria and refined *B*-factors. The final model was refined to *R* = 17.6% and *R*_free_ = 22.4%. Details of the data reduction and structure refinement are shown in Table 2.

Electron density could not be observed for the first two residues and the last 51 residues (missing C-terminus). Additional electron density in the active site was assigned to the cofactor FAD and dicoumarol. Validation of the structure was carried out with molprobity [30], yielding a Ramachandran plot with 97.3% of the residues in favored regions, 2.7% in allowed regions, and none in disallowed regions. Prediction of the biologically active form of NQO1 P187S (C-terminal truncation) was performed with the pisa server [31].

### Labeling of NQO1and NQO1 P187S with ^15^N

Instead of LB broth (Lennox), a minimal medium containing 6.8 g·L^−1^ Na_2_HPO_4_, 3 g·L^−1^ KH_2_PO_4_, 0.5 g·L^−1^ NaCl, 1 g·L^−1^ ^15^NH_4_Cl, 3 g·L^−1^ glucose, 1 μg·L^−1^ biotin, 1 μg·L^−1^ thiamin, 50 μg·mL^−1^ kanamycin and 1 mL of × 1000 microsalts [150 mm CaCl_2_, 20 mm FeCl_3_, 50 mm H_3_BO_3_, 150 μm CoCl_2_, 800 μm CuCl_2_, 1.5 mm ZnCl_2_, 15 μm (NH_4_)_6_Mo_7_O_24_·4H_2_O] was used for protein expression as described above.

### NMR spectroscopy

One-dimensional ^1^H-NMR spectra were recorded with a Bruker Avance III 500-MHz NMR spectrometer at 298 K (Bruker, Rheinstetten, Germany). All other NMR experiments were carried out with a Bruker Avance III 700-MHz NMR spectrometer, equipped with a cryogenically cooled, 5-mm TCI probe at 298 K. Samples containing 20–40 mg·mL^−1^ NQO1 or NQO1 P187S in 50 mm Hepes (pH 6.5) in 90% H_2_O and 10% D_2_O were used for NMR measurements. For ^15^N relaxation measurements, series of 10 interleaved, relaxation-edited, ^1^H/^15^N-HSQC spectra were recorded. The spectra were processed with nmrpipe [34] and analyzed with ccpnmr [35]. Rotational correlation times were calculated from the ^15^N *T*_1_/*T*_2_ ratios as previously described [36].

### Analytical size exclusion chromatography

Size exclusion chromatography was performed with a pre-packed Superdex-200 10/300 GL column (Pharmacia) equilibrated with a 50 mm Hepes (pH 7) and 150 mm NaCl buffer solution. Purified NQO1 and NQO1 P187S (100 μL of ~ 300 μm) were injected separately and eluted with a 0.5 mL·min^−1^ flow rate. Protein elution was monitored by following the absorption at 280 nm and 455 nm. The *A*_280 nm_ (mAU) values were normalized to 1 by dividing all the values by the maximum absorption value in the elution profile; all of the normalized values were further multiplied by a factor of 1000. The Superdex-200 column was calibrated with molecular mass standards according to the manufacturer’s instructions.

### Limited proteolysis

NQO1, apo-NQO1 and NQO1 P187S at 30 μm in 50 mm Hepes and 150 mm NaCl (pH 7.5) buffer were subjected to limited proteolysis at 37 °C by the addition of trypsin (Promega, Madison, WI, USA) to a final concentration of 2 μg·mL^−1^. The reaction was stopped after 5, 10, 20 and 40 min by adding SDS sample buffer to aliquots of the reaction mixture and immediately boiling at 95 °C for 10 min. FAD at 300 μm was added to the protein solution prior to the addition of trypsin (Fig. 9). The samples were analyzed by performing SDS-PAGE with precast gradient gels (Thermo Scientific, Waltham, MA, USA) (Fig. 7A) or with linear 12.5% polyacrylamide gels (Fig. 9).

### Maldi-TOF MS

Coomassie-stained protein gel bands (1 and 2 in Fig. 7A) were excised and destained by following standard in-gel digestion protocols. The cysteines were in-gel-alkylated and reduced with iodoacetamide and dithiothreitol, respectively. The proteins were in-gel-digested with trypsin at 37 °C. Peptide mixtures were extracted after trypsin digestion, and the mixture was desalted with ZipTip (Millipore, Darmstadt, Germany). The purified peptides were spotted onto a MALDI target plate together with matrix α-cyano-4-hydroxycinnamic acid, and the spectra were recorded on a Micromass TofSpec 2E in reflectron mode at an accelerating voltage of +20 kV. The instrument was calibrated with a poly(ethylene glycol) mixture (Sigma-Aldrich, St Louis, MO, USA). ProteoMasS ACTH Fragment 18–39 (Sigma-Aldrich, St Louis, MO, USA) was used as the peptide calibration standard for the instrument. The spectra were analyzed with masslynx 4.1, and peptide mass profiles were assigned.

## Figures and Tables

**Table 1:** The reductive half-reaction of NQO1 P187S is compromised, thereby affecting its ability to accept electrons from NAD(P)H. Bimolecular rate constants (m^−1^·s^−1^) were determined for NQO1 proteins with NADH or NADPH as reducing agent. The absorption change of protein-bound FAD at 445 nm was monitored in a stopped-flow instrument at 4 °C.

Protein	NADH *k*_red_ (m^−1^·s^−1^)	NADPH *k*_red_ (m^−1^·s^−1^)
NQO1	(3.5 × 10^6^) ± (1.0 × 10^6^)	(5.7 × 10^6^) ± (2.7 × 10^6^)
NQO1 P187S	(1.2 × 10^4^) ± (1.7 × 10^3^)	(8.5 × 10^4^) ± (1.8 × 10^6^)
NQO1 Δ50	(4.7 × 10^3^) ± (0.7 × 10^3^)	(1.3 × 10^4^) ± (0.1 × 10^4^)
NQO1 P187S Δ50	(3.2 × 10^3^) ± (0.2 × 10^3^)	(8.2 × 10^4^) ± (0.2 × 10^4^)

**Table 2:** Crystallographic data and refinement statistics for the NQO1 P187S and NQO1 P187S Δ50 crystal structures (50 amino acids truncated at the C-terminus).

	NQO1 P187S	NQO1 P187S Δ50
Data collection		
X-ray source	ESRF ID-29	ELETTRA XRD1
Wavelength (Å)	0.9724	1.1354
Temperature (K)	100	100
Space group	I222	P4_1_2_1_2
Cell dimensions		
*a, b, c* (Å)	104.17, 104.56, 118.57	51.05, 51.05, 169.04
Resolution (Å)	26.74–2.69	33.81–2.20
High-resolution shell	2.84–2.69	2.26–2.20
Total no. of reflections	114 922 (16 147)	144 289 (7592)
Unique no. of reflections	18 149 (2523)	12 127 (843)
Multiplicity	6.3 (6.4)	11.9 (9.0)
Completeness (%)	99.5 (97.0)	99.8 (98.0)
*R*_p.i.m._ (%)	6.4 (31.4)	3.5 (21.0)
*R*_meas_ (%)	16.0 (80.7)	12.4 (65.5)
*I/σ* (*I*) average	9.3 (2.4)	18.3 (3.2)
Refinement		
Resolution (Å)	46.22–2.69	33.20–2.20
High-resolution shell	2.83–2.69	2.42–2.20
*R*_work_	0.1847 (0.3102)	0.1755 (0.2292)
*R*_free_	0.2074 (0.3448)	0.2239 (0.2955)
No. of atoms	4531	2004
Protein	4316	1787
Cofactor/substrate	208	78
Water	7	139
Protein residues	541	222
*B*-factors (total)		
Protein (Å^2^)	41.9	34.1
Cofactor/substrate (Å^2^)	44.2	30.3
Water (Å^2^)	38.0	37.8
All atoms (Å^2^)	42.0	34.2
Rmsds		
Bond lengths (Å)	0.003	0.004
Bond angles (°)	0.719	0.895
Ramachandran	0	0
outliers (%)		

Values in parentheses are for the highest-resolution shell.

## Abbreviations

ITC: isothermal titration microcalorimetry
NQO1: NAD(P)H:quinone oxidoreductase 1
PDB: Protein Data Bank
SNP: single-nucleotide polymorphism
WT: wild type
